# Endophytic Fungi *Piriformospora indica* Mediated Protection of Host from Arsenic Toxicity

**DOI:** 10.3389/fmicb.2017.00754

**Published:** 2017-05-10

**Authors:** Shayan Mohd, Jagriti Shukla, Aparna S. Kushwaha, Kapil Mandrah, Jai Shankar, Nidhi Arjaria, Prem N. Saxena, Ram Narayan, Somendu K. Roy, Manoj Kumar

**Affiliations:** ^1^Environmental Toxicology Group, CSIR-Indian Institute of Toxicology ResearchLucknow, India; ^2^Academy of Scientific and Innovative Research (AcSIR), CSIR-IITR CampusLucknow, India; ^3^Regulatory Toxicology Group, CSIR-Indian Institute of Toxicology ResearchLucknow, India; ^4^Electron Microscope facility, CSIR-Indian Institute of Toxicology ResearchLucknow, India; ^5^Central Confocal Facility, CSIR-Indian Institute of Toxicology ResearchLucknow, India

**Keywords:** arsenic, arsenic toxicity, abiotic stress tolerance, endophytic fungi, symbiosis, plant microbe interactions, bioremediation, hyper-colonization

## Abstract

Complex intercellular interaction is a common theme in plant-pathogen/symbiont relationship. Cellular physiology of both the partners is affected by abiotic stress. However, little is known about the degree of protection each offers to the other from different types of environmental stress. Our current study focused on the changes in response to toxic arsenic in the presence of an endophytic fungus *Piriformospora indica* that colonizes the paddy roots. The primary impact of arsenic was observed in the form of hyper-colonization of fungus in the host root and resulted in the recovery of its overall biomass, root damage, and chlorophyll due to arsenic toxicity. Further, fungal colonization leads to balance the redox status of the cell by adjusting the antioxidative enzyme system which in turn protects photosynthetic machinery of the plant from arsenic stress. We observed that fungus has ability to immobilize soluble arsenic and interestingly, it was also observed that fungal colonization restricts most of arsenic in the colonized root while a small fraction of it translocated to shoot of colonized plants. Our study suggests that *P*. *indica* protects the paddy (*Oryza sativa*) from arsenic toxicity by three different mechanisms viz. reducing the availability of free arsenic in the plant environment, bio-transformation of the toxic arsenic salts into insoluble particulate matter and modulating the antioxidative system of the host cell.

## Introduction

Cellular metabolism shapes the phenotypes of cells/organism. Kinetic changes in cellular metabolism provide flexibility and robustness for adaptability in response to environmental cues. The changes are much more evident when cells are challenged either with toxic doses of chemicals or in response to infectious virus, fungi, and bacteria. The interaction becomes much more complex when both the biotic and abiotic factors act in collusion. Heavy anthropological and geological activities are a major contributor of toxic products in nature. (Nordstrom, 2002; Jang et al., 2007). The toxicants, in turn, pose a serious risk to health and environment when they end up at the top of the food chain after being taken up by the plants. Toxicity of the harmful chemical largely depends on the geological and biological activities that determine the free availability of the toxic ions. Metal and metalloid toxicity is a serious environmental and health issue. While technological advancement is the need of the hour, new innovations are a must. Mechanistic understanding of bioremediation of heavy metals and metalloids can provide new tools and resources for the proper management of environmental toxicants. One should understand the process of detoxification of elements as these elements cannot be degraded. Major mechanisms of detoxification of the elements are only possible by compartmentalization and biotransformation into an inactive form of element or safe excretion by the cell or organism (Nies et al., 1989; Nies, 1999). Agriculture land contaminated with arsenic is one of the major problems of developing countries including India, Bangladesh, and China; where overexploitation of groundwater containing arsenic, is in practice for cultivation. Irrigation practices with groundwater increases surface arsenic and accumulation in crops that is finally consumed by animals and humans leading to several health complications such as skin lesions, neurological impairment, and cancer (Smedley and Kinniburgh, 2002; Huq et al., 2006; Ratnaike, 2006; Wei et al., 2013; Ramos-Chavez et al., 2015; Singh et al., 2015; Wiwanitkit, 2015; Wu et al., 2015). In the plants arsenic mainly interferes with photosynthesis and reduces transpiration efficiency. Impact and accumulation of arsenic on the plants generally associated with the genotype which decide the sensitivity and response of arsenic on the metabolism of plant (Finnegan and Chen, 2012).

Plants modulate the biochemical and molecular response upon arsenic exposure to minimize the toxic effects on the cell metabolism viz. expression pattern of phosphate and hexose transporters, antioxidative enzyme system, antioxidant metabolite pools, glutathione metabolism, phytochelatins (PC), and vacuolar PC-As transporters (Finnegan and Chen, 2012; Tripathi P. et al., 2012; Tripathi R. D. et al., 2012; Chen et al., 2017). However, these biochemical and molecular changes varies with the species to species of economically important crops which demarcate the tolerant and sensitive variety with broad range of arsenic bioaccumulation (Rai et al., 2011). Bioaccumulation restriction of toxic arsenic might be the solution to prevent it from entering the food chain. Several microorganisms have been used for bioremediation of such toxic metals and metalloids. However, their utilization is limited to the specific niche from where these microbes were isolated or their axenic cultures not available such as in the case of mycorrhiza. Introduction into other niche destabilizes the micro-environment and biogeochemical cycles, therefore, an alternative strategy is needed to deal with metal bioremediation.

Previous work suggest that the specific fungal strains in the soil reduces the negative effects of arsenic on the plants however it is unknown that how this phenomenon of tolerance against arsenic is mediated by fungi (Srivastava et al., 2012; Tripathi et al., 2013; Spagnoletti and Lavado, 2015). We hypothesized that the fungal induced tolerance of host cell against arsenic might be an innovative solution to the problem. We used *Piriformospora indica* a root colonizing endophytic fungus which attributes several beneficial traits to their host with a wide range of biotic and abiotic stress tolerance, growth and biomass yield promotion (Kumar et al., 2009, 2011; Yadav et al., 2010; Jogawat et al., 2013; Johri et al., 2015) to induce arsenic resistance by reprogramming the metabolism of rice plant and reduce arsenic load in the host.

## Materials and methods

Plant, fungal culture, and growth conditions: Rice (*Oryza sativa* L. IR64) seeds were surface sterilized for 2 min in 70% ethanol followed by 10 min in a NaClO solution (0.75% Cl). Seeds were finally washed six times with sterile water and further dH_2_O at 60°C for 5 min to eliminate naturally occurring microbes that may have been in or on the rice seed as described previously (Kumar et al., 2009). Seeds were germinated on water-agar plates (0.8% Bacto Agar; Difco, Detroit, MI) at 37°C in the dark. *P. indica* was cultured on *Aspergillus* minimal media for 8 days (Hill and Kafer, 2001; Yadav et al., 2010). For colonization, radicle of seedling plants were transferred into sterile spore suspension (10^6^ spores per ml) for 2 min and further transferred to water agar plate for 1 day, while in case of control plants autoclaved spore suspension was used. This procedure of inoculation of spore gives a similar and uniform level of colonization in root of every inoculated plant. Further, seedlings were transferred to closed lid jars for hydroponic culture. Rice plants were grown in a growth chamber under controlled temperature and humidity (32°C/70%). One-quarter strength of hydroponic solution (Kamachi et al., 1991) was changed weekly with 10 ml fresh solution containing arsenic (100 μM sodium arsenate; Ahsan et al., 2008) respectively in each jar and removed solution was kept for arsenic analysis.

In order to study bioprotection offered by *P. indica* against arsenic toxicity all plants were initially grown for 3 days and subsequently following types of sets were used. (Set-1), rice plants were grown till 25 days without any fungus were used as a control; (Set-2), rice plants were inoculated only with *P. indica* at day zero and grown till 25 days; (Set-3), rice plants treated with sodium arsenate at day zero and grown till 25 days; (Set-4), rice plants were colonized with *P. indica* and at day 3 treated with sodium arsenate and grown for total 25 days (Delayed treatment); (Set-5), rice plants inoculated with *P. indica* and sodium arsenate at day zero and grown for total 25 days (Simultaneous treatment); (Set-6), *P. indica* was inoculated at day 3 after treatment with sodium arsenate (this time point is considered as day zero) and grown for next 25 days (Early treatment). For this experiment, control plants and treatment were set accordingly. Plants were harvested at different time periods and carefully washed and rinsed in de-ionized autoclaved water and weighed. Plant samples were stored in water for 1 h to study colonization, however for superoxide accumulation, arsenic uptake, and enzyme assays study fresh samples were used. Growth promoting effect of fungus was checked by measuring the dry weight. To measure dry weight, plant materials after harvesting were kept at 100°C for 72 h in a hot air oven. All experiments were done independently in triplicates and for each case 15 seedlings were used per jar in triplicates.

### Fungal growth condition and arsenic treatment

Fungus was grown in minimal media with different concentration of sodium arsenite (As III) and sodium arsenate (As V) at 0, 1, 1.5, 2, 2.5 mM. After 1 week of growth mycelia was filtered and washed 10 times with autoclaved MQ water and fresh biomass was taken after drying the excess moisture on blotting paper.

### Fungal colonization analysis

To study colonization, 10 root samples were selected randomly from the rice root. Samples were softened in 10% KOH solution for 15 min and acidified with 1N HCl for 10 min and finally stained with 0.02% Trypan blue overnight (Kumar et al., 2009; Jogawat et al., 2013). Samples were destained with 50% Lacto-phenol for 1–2 h prior to observation under light microscope (Zeiss Microscope, Germany). The distribution of chlamydospores within the root was taken as an index for studying colonization. Percent colonization was calculated for the inoculated plants according to the method described previously (Mcgonigle et al., 1990). To check the effect of arsenic toxicity on the colonization pattern of endophyte, root samples examined for the presence of *P. indica* within the root tissues of plant stained with Calcofluor White stain. Samples were fixed prior to observation under a confocal microscope (Leica TCS CS2 confocal Microscope). For real-time PCR comparison, 0.15 g of root tissues of the each sample was used to isolate total genomic DNA by the CTAB method. PCR reactions were carried out with 1 μg of genomic DNA as template and specific primers using sybr green. Specific primers for EF-1-alpha (*tef*) gene (AJ249912) of *P. indica* PitefFOR (5′–TCGTCGCTGTCAACAAGATG-3′) and PitefREV (5′–GAGGGCTCGAGCATGTTGT-3′) and actin gene (AB047313.1) of rice plant OsActinF (5′-GCCGTCCTCTCTCTGTATGC-3′) and OsActinR (5′-GACGAAGGATAGCATGGGGG- 3′) were used.

### Antioxidant enzyme activities in rice plants

In order to know the impact of colonization of *P. indica* on antioxidative system of plant during arsenic stress, antioxidative enzyme activities were also checked in the presence, absence, and delayed treatment of arsenic. For this purpose, all the experiments and conditions were kept same as described in the previous section. Protein isolation was done as described previously with some modifications (Kumar et al., 2009), fresh root and shoot tissue were homogenized at 4°C in an ice-chilled mortar with liquid N_2_in QB buffer [without DTT (1,4-Dithiothreitol) for SOD, CAT, and GST assay] with 50 mg polyvinyl pyrrolidone (PVP) per gram tissue (for GR assay). Crude homogenates were centrifuged at 15,000 × g for 15 min at 4°C and the supernatant fractions were kept frozen at −20°C. Protein contents were determined by Bradford method using BSA as standard (Bradford, 1976).

#### SOD assay

In this case, activity was monitored according to the method described previously (Roth and Gilbert, 1984). One milliliter of reaction mixture contains 50 mM sodium phosphate buffer (pH 7.8), 100 μM EDTA, with 20 μl of enzyme extract, and 10 mM of pyrogallol. The enzyme activity (U/mg Protein) was calculated by scanning the reaction mixture for 120 s (60 s interval) at 420 nm.

#### CAT assay

Catalase activity was assayed by measuring the initial rate of H_2_O_2_ disappearance using the method described previously (Beers and Sizer, 1952). One milliliter of catalase assay reaction mixture contains 0.05 mM sodium phosphate buffer (pH 7.0) with 20 μl of enzyme extract and 1 mM of H_2_O_2_. The decrease in H_2_O_2_ was followed by a decline in optical density at 240 nm, and the activity (U/mg protein) was calculated using the extinction coefficient of 40 mM cm^−1^ for H_2_O_2_.

#### GST assay

For the measurement of GST activity, 1 ml of reaction mixture contained 0.1 M sodium phosphate buffer (pH 6.5) with 20 μl of enzyme extract and 2% CDNB (1-chloro-2,4-dinitrobenzene). The enzyme activity (U/mg protein) was calculated by scanning the reaction mixture for 180 s (60 s interval) at 340 nm. GST activity was monitored as described previously (Habig et al., 1974).

#### GR assay

The activity (U/mg protein) was determined by the oxidation of NADPH at 340 nm with an extinction coefficient of 6.2 mM cm^−1^, as described previously (Nordhoff et al., 1997). The reaction mixture was composed of 100 mM potassium phosphate buffer (pH 7.8), 2 mM EDTA, 0.2 mM NADPH and 0.5 mM glutathione (oxidized form, GSSG), and 10 μl of enzyme extract (total reaction mixture 1 ml). The reaction was initiated by the addition of NADPH at 25°C.

### Estimation of H_2_O_2_ production

The H_2_O_2_ content from rice seedlings was measured as described earlier (Junglee et al., 2014). One gram tissue was extracted with 5 ml of TCA (0.1%, w/v) at 48°C and homogenate was centrifuged at 12,000 g for 15 min. To 0.5 ml supernatant, 0.5 ml of 0.05 M sodium phosphate buffer (pH 7.0) and 1 ml of 1 M potassium iodide solution were added. The absorption of the mixture was measured at 390 nm using UV-visible spectrophotometer (Thermo Fisher Scientific). The H_2_O_2_ content was determined using an extinction coefficient (ϵ) of 0.28 mM^−1^ cm^−1^ and expressed as mg g^−1^ fresh weight.

### Estimation of chlorophyll

Chlorophylls and carotenoids were isolated from leaves by homogenization in liquid nitrogen and subsequent threefold extraction with 80% acetone (v/v). After centrifugation for 5 min at 1,500 × g, the absorbance of the supernatant was measured at 663.6, 646.6, and 440.5 nm (Porra et al., 1989). Leaf samples were also extracted with 1% (w/v) HCl in methanol, and the anthocyanin contents were assayed spectrophotometrically. The relative amounts of anthocyanins were expressed by [A_530_–0.333A_657_] m^−2^ (Mancinelli et al., 1975). Absorbance was measured with a UV-visible spectrophotometer (Thermo Fisher Scientific).

### Estimation of proline

Proline content in leaf tissues was determined by the ninhydrin method (Jogawat et al., 2013) using UV–vis spectrophotometer (Thermo Fisher Scientific). It was calculated with using standard curve at 520 nm and expressed as mmol per gram fresh weight.

### Dithizone test for the accumulation of arsenic in plant and fungus

To analyze the accumulation of arsenic in plant dithizone test was performed. 3 mg of dithizone was dissolved in 6 ml of acetone; then 2 ml of distilled water and 1–2 drops of pure acetic acid were added. Plant samples were gently washed in distilled water and incubated in this solution for 1–24 h in the dark. After staining the shoots were observed for accumulated metal (Turnau and Wierzbicka, 2006).

### Analysis of zeta potential of fungal cell wall

*P. indica* Fungus was grown in minimal media with different concentration of As (III) and As (V). After 1 week of growth, mycelia was filtered and washed 10 times with autoclaved MQ water and three times with PBS. Mycelia was macerated in tissue grinder and again washed (three times) with autoclave MQ water. Finally, macerated mycelia was suspended in MQ water and analyzed for zeta potential using ZetaSizer 3.0 (Malvern). For adsorption studies of arsenic on the fungal cell wall, 1 g of cell wall was incubated in 100 ml of arsenic solution for 60 min. and cell wall material was washed with PBS three times. These samples were digested with acids for arsenic analysis. Adsorbed arsenic analysis was done by colorimetry in triplicates, independently (Pillai et al., 2000). Percent adsorption was calculated using total supplied arsenic and adsorbed arsenic on the cell wall.

### Arsenic estimation in plant materials

The concentration of arsenic ions in plant materials was determined by Atomic Fluorescence Spectrophotometer (AFS, PG Instruments AF-420) equipoise with an auto sampler, hydride generator, and high-intensity hollow cathode lamps. For this 500 mg of plant materials and 100 mg of fungal materials were acid digested at 80°C and further diluted in 1 N nitric acid and used as a sample for arsenic estimation. Independent triplicate was used to calculate arsenic in plant materials.

### Transmission electron microscope, scanning electron microscope, and EDAX analysis (TEM, SEM, and EDAX analysis)

Fungus was grown in minimal media with sodium arsenate (As V) at 0.1 and 0.5 mM concentration while control was grown without arsenic. After 3 days of growth, mycelia was filtered and washed 10 times with autoclaved MQ water. Further, fungal mycelium of control and treated were washed with 1XPBS (pH 7.2) and fixed in 2.5% glutaraldehyde prepared in phosphate buffer (pH 7.4) for 2 h at 4°C. Cells were washed three times with 0.1 mM phosphate buffer and post fixed in 1% Osmium tetroxide for 4 h. Fixed cells were washed with phosphate buffer, dehydrated in acetone series (15–100%), and embedded in Araldite-DDSA mixture (Ladd Research Industries, USA, Burlington). After baking at 60°C, block was cut (60–80 nm thick) by an ultramicrotome (Leica EM UC7) and sections were stained with Uranyl acetate and Lead citrate. Analysis of sections was done under FEI Tecnai G2 spirit twin transmission electron microscope equipped with Gatan digital CCD camera (Netherlands) at 80 KV. The confirmation of insoluble precipitates as arsenic was done by SEM (Scanning electron microscope) equipped with EDAX.

### Statistical analysis

All graphs were created and statistical calculations were performed using Microsoft Excel 2007. The significance of the data obtained was checked by Student's *t*-test using the program SPSS Statistics 20.0 (IBM USA).

## Results

### Endophytic colonization under arsenic stress

**Figure 1:**
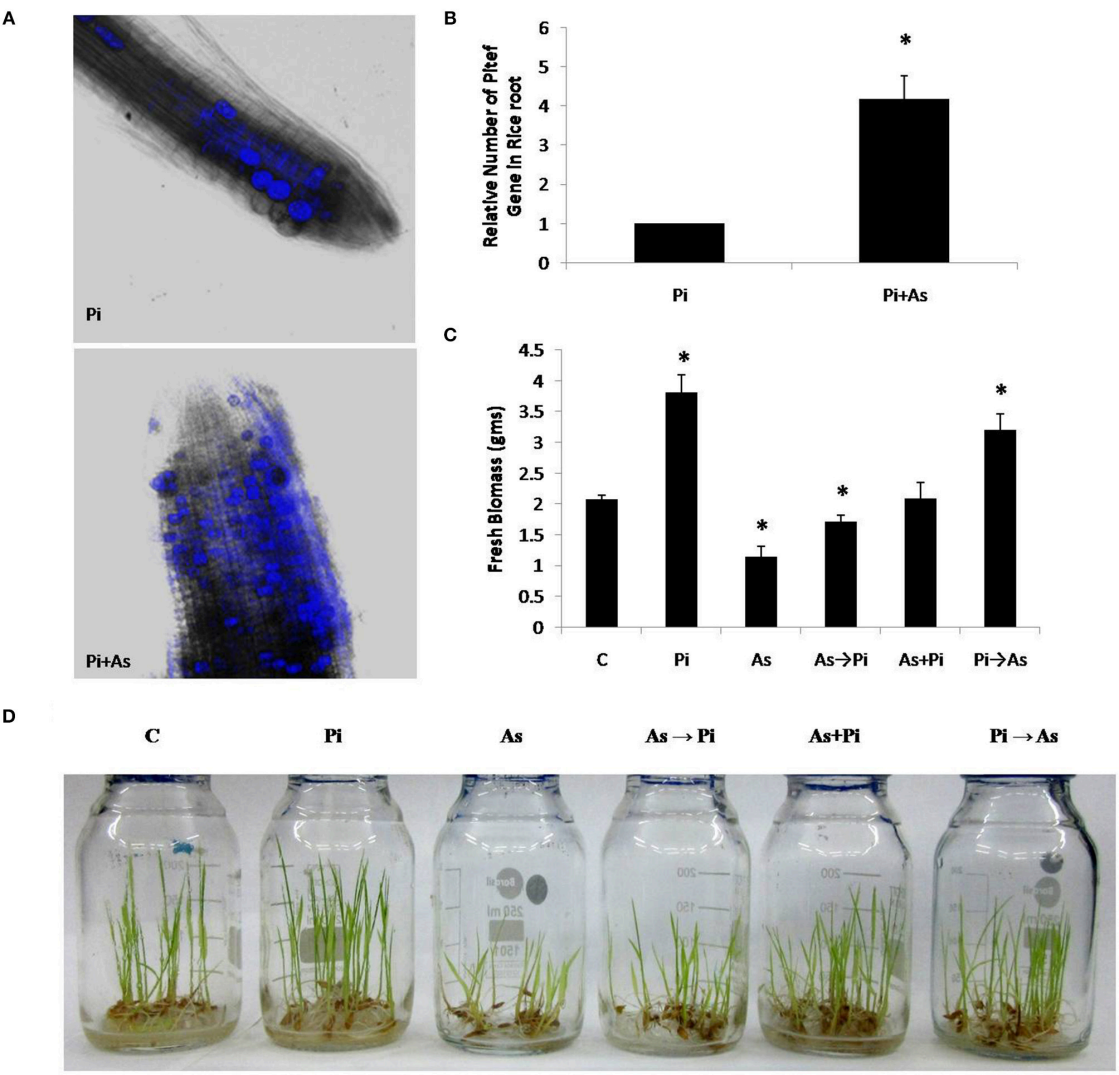
**Colonization of ***P. indica*** under arsenic stress. (A)** Confocal microscope and **(B)** real time PCR analysis was done to characterize colonization of fungus *P. indica* under arsenic stress 100 μM sodium arsenate Pi+As after 25 dai (days after inoculation) in the rice root, the hyper-colonization was observed than the untreated control Pi. **(C,D)** Alternate and simultaneous inoculation of *P. indica* was done to show the pattern of recovery at the level of biomass of rice plant to the arsenic-treated plants. **(C)** Rice plants grown for 25 days without any fungus were used as a control; Pi, rice plants inoculated with *P. indica* alone at day 0 and grown for 25 days; As, rice plants treated with arsenic at day 0 and grown for 25 days; As→Pi, rice plants first treated with arsenic at day 0 and at day 3 inoculated with *P. indica* and grown for a total of 25 days; Pi+As, rice plants inoculated simultaneously with both fungi and arsenic at day 0 and grown for 25 days; Pi→As, rice plants first inoculated with *P. indica* at day 0 and at day 3 treated with arsenic and grown for a total of 25 days. Maximum recovery in the biomass is maximum when already colonized plant was treated with arsenic. Asterisks show values significantly different from those of the controls (*P* < 0.05).

In the colonization study, it was observed that pre-colonization of *P*. *indica* in paddy plant is a time dependent process and is unaffected by arsenic salt concentration that is otherwise toxic to plants. About 50–60% colonization was also observed at 2 days after inoculation (dai). Colonization increases up to 75% at 5 dai and is maintained during the study however colonization more than 75% was not observed (Table 1). No adverse effect of arsenic at given concentration on fungal colonization and sporulation was seen when *P. indica* was inoculated after arsenic treatment. Fungal colonization was confirmed by real-time PCR analysis and intracellular pear-shaped chlamydospores (Figures 1A,B). Real-time PCR analysis shows that four times higher fungal load in the As treated root than untreated roots. A similar pattern of colonization was also observed by confocal microscopy in the 25 day old roots (Figures 1A,B; Supplementary Figure 1).

**Table 1:** **Effect of arsenic on colonization of root**.

**S. No**.	**Dai (days after inoculation)**	**Percent colonization (control plant)**	**Percent colonization (as treated plant)**
1	1	~10	~10
2	2	~60	~60
3	3	~75	~75
4	4	~75	~75
5	5	~75	~75

*The chlamydospores distribution within the root was considered as an indicator for presence of fungus and this was applied to study colonization. Percent colonization was calculated for the inoculated plants according to the method described previously (Mcgonigle et al., 1990)*.

### Endophyte recovers plant growth from arsenic toxicity

A distinct morphological and biomass changes observed in plant colonized with *P. indica* as compared to non-colonized plants (used as a control) (Figures 1C,D). Plants treated with arsenic shows shoot stunting and poor growth as compared to control plants (As; Figure 1D). We found that plants colonized with *P. indica* first and treated with arsenic at day 3 showed increased overall growth as compared to arsenic treated non-colonized plant that was equivalent to control plants (Pi→As; Figure 1D). Similar morphological patterns were also observed in delayed fungal inoculated treated plants and it showed improved root and shoot growth (As→Pi; Figure 1D).

A significant increase was also observed in biomass i.e., 1.7 fold in 25 days old paddy plants colonized with *P. indica* as compared to non-colonized plants (*P* < 0.05) (Pi; Figure 1C). Plants treated with arsenic showed a 1.72 fold decrease in dry weight of 25 days old rice plants as compared with control plants (non-colonized) (*P* < 0.05) (As; Figure 1C). Inoculation of *P. indica* at day 3 after arsenic treatment resulted in improved biomass yield i.e., 1.8 fold increase in dry weight as compared to plants treated with arsenic and is comparable to control plants (*P* < 0.05) (Figure 1C). In another case, where plants treated simultaneously with *P. indica* and arsenic at day 0 showed 1.16 fold increases in dry weight in comparison to control (plants without fungus and arsenic treatment) (Figure 1C). Our study shows that *P. indica* colonization promoted overall plant growth even in presence of toxic dosage of arsenic as compared to control plant (Figures 1C,D).

**Figure 2:**
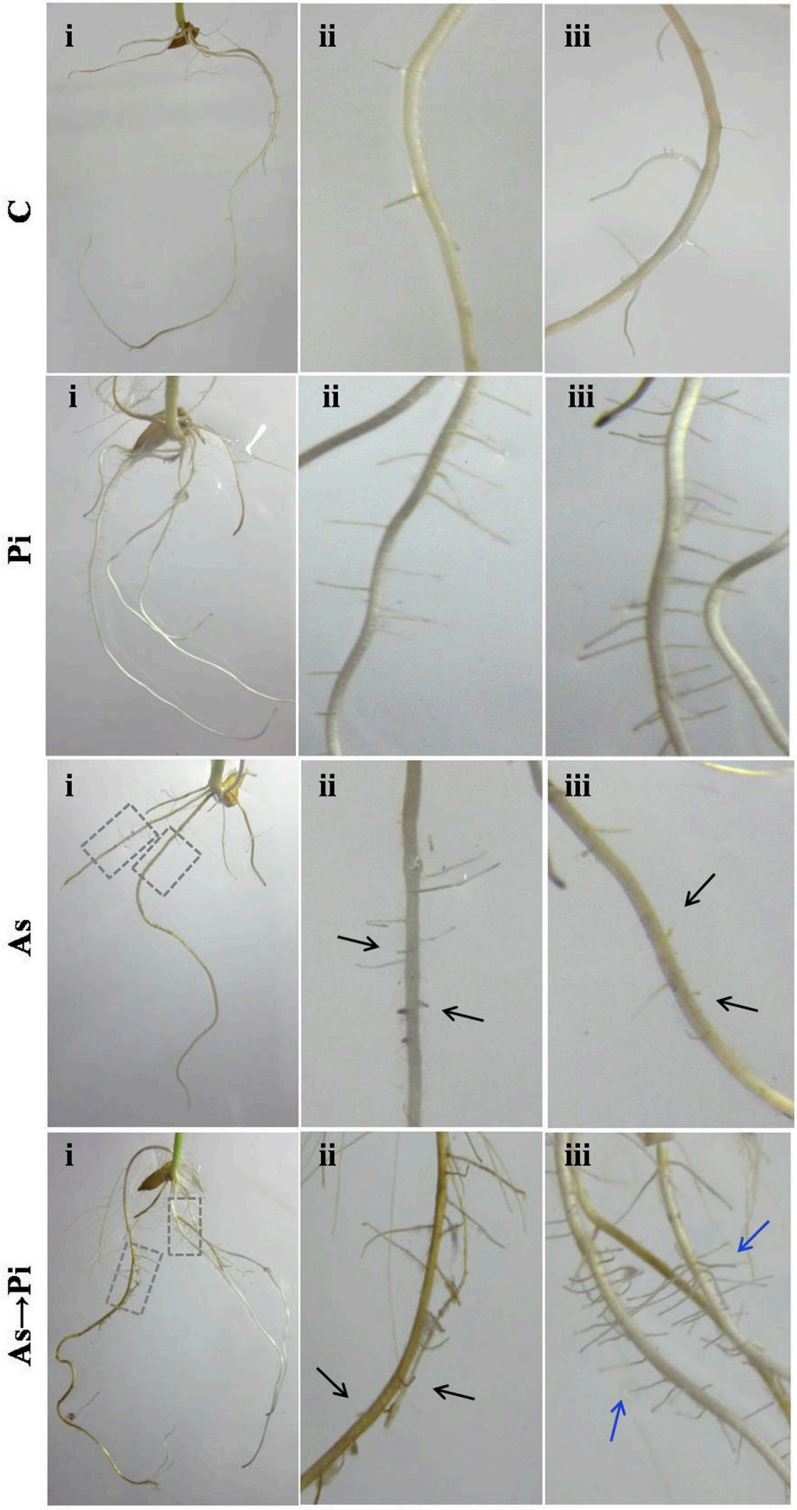
*****P. indica*** recovers root damage by arsenic**. Inoculation of *P. Indica* to the arsenic-treated plants induced new healthy root formation. Damaged root branches in arsenic-treated plants As are shown by black arrows, and recovery in root branches in *P. indica* colonized plants (As→Pi) are shown by blue arrows. C, rice plants grown for 25 days without any fungus were used as a control; Pi, rice plants inoculated with *P. indica* alone at day 0 and grown for 25 days; As, rice plants treated with arsenic at day 0 and grown for 25 days; As→Pi, rice plants first treated with arsenic at day 0 and at day 3 inoculated with *P. indica* and grown for a total of 25 days.

Root morphology is an important health recovery parameter to analyze arsenic toxicity. Arsenic treated plants are badly damaged at the root level and show a poor growth of primary and almost nil secondary roots as compared to control plant (As; Figure 2). Colonization of *P. indica* shows an increase in growth of primary root and number of secondary root as compared to control plant (Pi; Figure 2). *P. indica* inoculation in arsenic-treated plants improved root growth and number of secondary root. Further new roots emerged bearing healthy secondary roots (As→Pi; Figure 2).

### *P. indica* protects rice plant from arsenic toxicity

**Figure 3:**
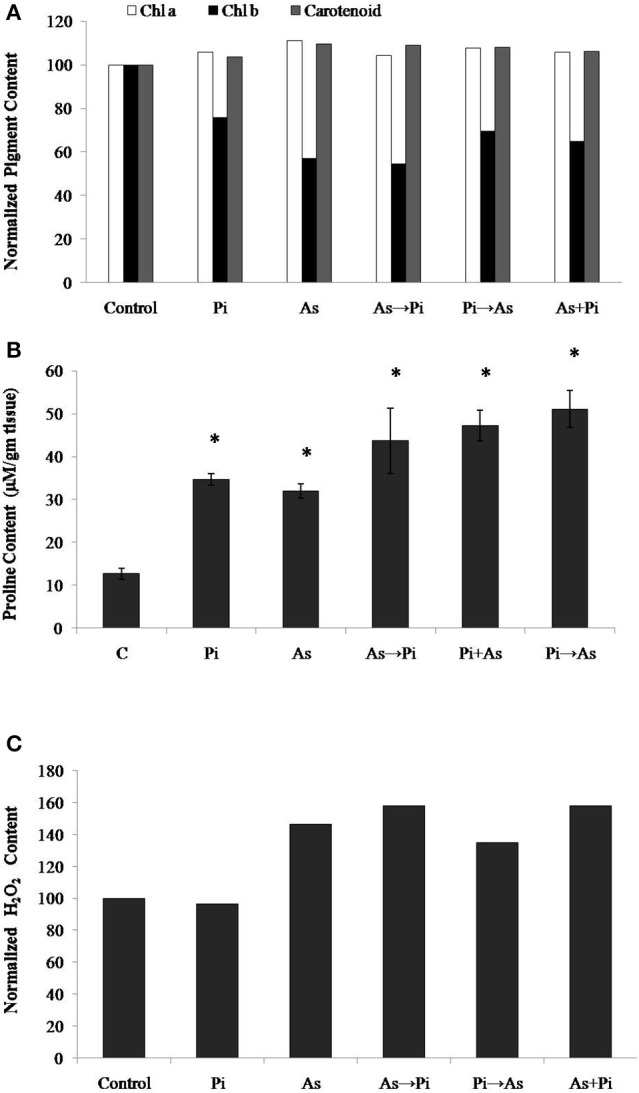
**Biochemical changes induced by ***P. indica*** may protect plant from arsenic-induced toxicity. (A)** Normalized chlorophyll a, b, and carotenoids content; **(B)** Proline content; and **(C)** H_2_O_2_ content in the plants alternate and simultaneously treated with *P. indica* and arsenic. All experimental conditions were the same as described for Figure 1. Asterisks show values significantly different from those of the controls (*P* < 0.05).

Arsenic treatment decreased total chlorophyll content by 10% and chlorophyll-b content by 43% while 11 and 9% increase was observed for chlorophyll-a and carotenoids respectively as compared to control (Figure 3A). However, *P. indica* increases the pigment contents upon colonization both in presence and absence of arsenic as compared to arsenic-treated plants (Figure 3A). Arsenic treatments increased the proline contents in rice seedlings indicating the influence of enhanced oxidative stress. The proline contents were increased by about 1.5–3 times higher on arsenic treatment while colonization of endophyte increases the proline content about two to five times higher than the control throughout the study. Similarly, the delayed and alternate treatment of arsenic and endophyte increases proline contents up to five times and was equivalent to untreated colonized plant (Figure 3B). Long-term induction of proline in colonized plant is a characteristic phenomenon of *P. indica* colonization (Jogawat et al., 2013).

The H_2_O_2_ level is a measure of oxidative environment in the rice plant upon exposure of biotic and abiotic stresses. H_2_O_2_ contents in arsenic-treated plant was 50% higher as compared to control, however, it was 4% lower in fungus colonized plant. In case of arsenic treatment before colonization (As→Pi) and simultaneous treatment of arsenic and endophyte (As+Pi), H_2_O_2_ level was measured 10% higher as compared to arsenic-treated plants in both cases (Figure 3C) while in case of arsenic treatment after colonization (Pi→As), H_2_O_2_ level was measured 8% lower as compared to arsenic-treated plant.

### *P. indica* regulates anti-oxidative enzyme in rice plant

**Figure 4:**
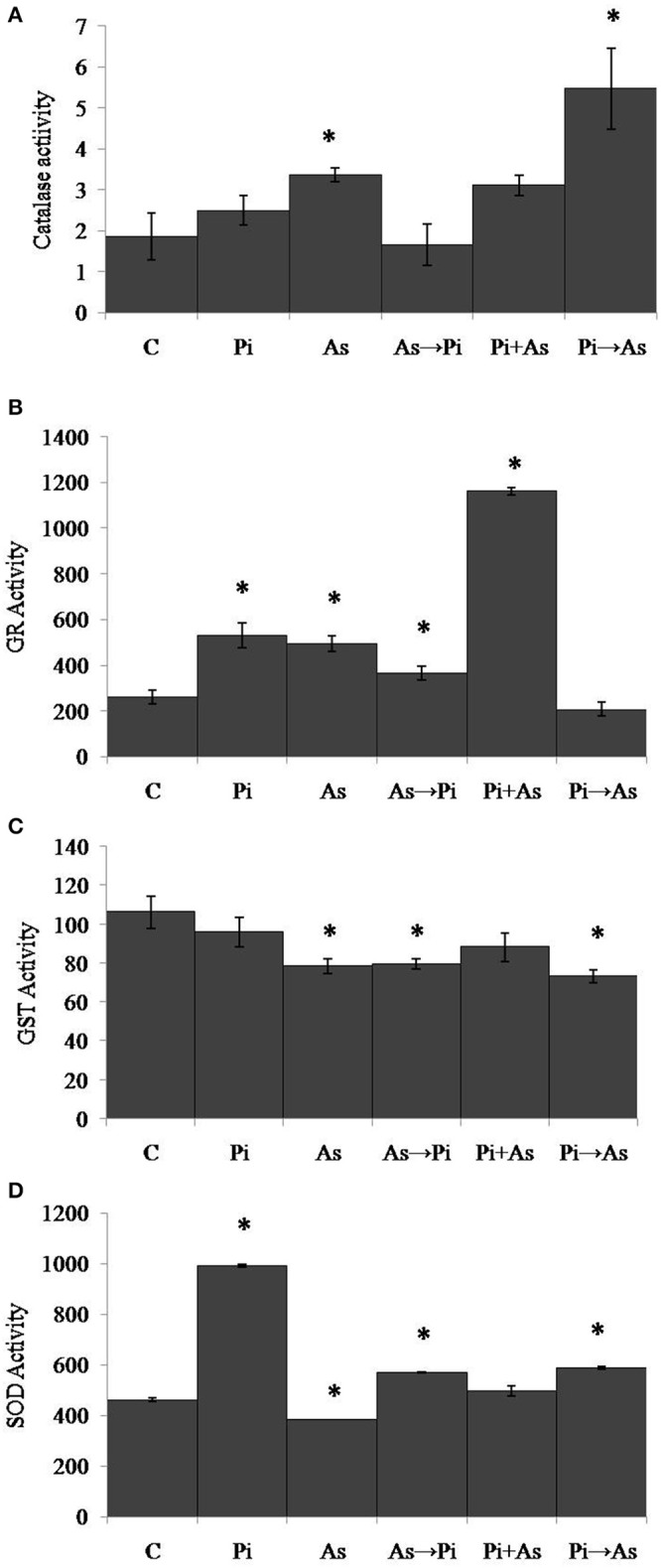
**Modulation of redox enzyme in rice root after treatment of ***P. indica*** and arsenic works as a defense against oxidative stress. (A)** CAT, **(B)** GR, **(C)** GST, and **(D)** SOD specific activities compared with those of control plants **(C)**; asterisks show values significantly different from those of the controls (*P* < 0.05). All experimental conditions were the same as described for Figure 1.

To analyze the effect of arsenic and fungus on the redox immunity of plant, antioxidative enzyme activity was measured. Exposure to arsenic increased catalase (CAT) activity by 1.8 fold as compared to non-colonized plant root (*P* < 0.05) (Figure 4A). Under similar conditions the activity of glutathione reductase (GR) was increased significantly (1.9 fold) (Figure 4B). Glutathione-S-Transferase (GST) and Super Oxide Dismutase (SOD) activity decreased significantly as compared to control plants (*P* < 0.05) (Figures 4C,D). In the case of plants colonized with *P. indica*, a maximum of 1.3 fold increased activity was observed for CAT as compared to non-colonized plants. Under similar conditions, GR and SOD activities were found to be increased by 2 and 2.1 fold. No significant change in activity was observed for GST (Figures 4B–D). Inoculation of *P*. *indica* in plants treated with arsenic (As→Pi) resulted decrease in enzyme activity for CAT and GST while increase in activity up to 1.4 and 1.2 fold for GR and SOD respectively than control plants and were found significant (*P* < 0.05) (Figures 4A–D). In the case of simultaneous treatment of As and fungus, CAT and GR activities were observed 1.7 and 4.4 fold higher than control plants while an insignificant decrease was observed in case of GST and no change in activity was observed for SOD. Similarly, in case of plants previously colonized with *P. indica* and later treated with arsenic (Pi→As), CAT and SOD activities were significantly higher than control and As treated plants (Figures 4A,D) while significantly decrease in activity was recorded for GR and GST as compared to control (Figures 4B,C). Interestingly, decrease was observed in GST activity than control plants in all cases of alternate and simultaneous treatment of As and fungus (Figure 4C).

**Figure 5:**
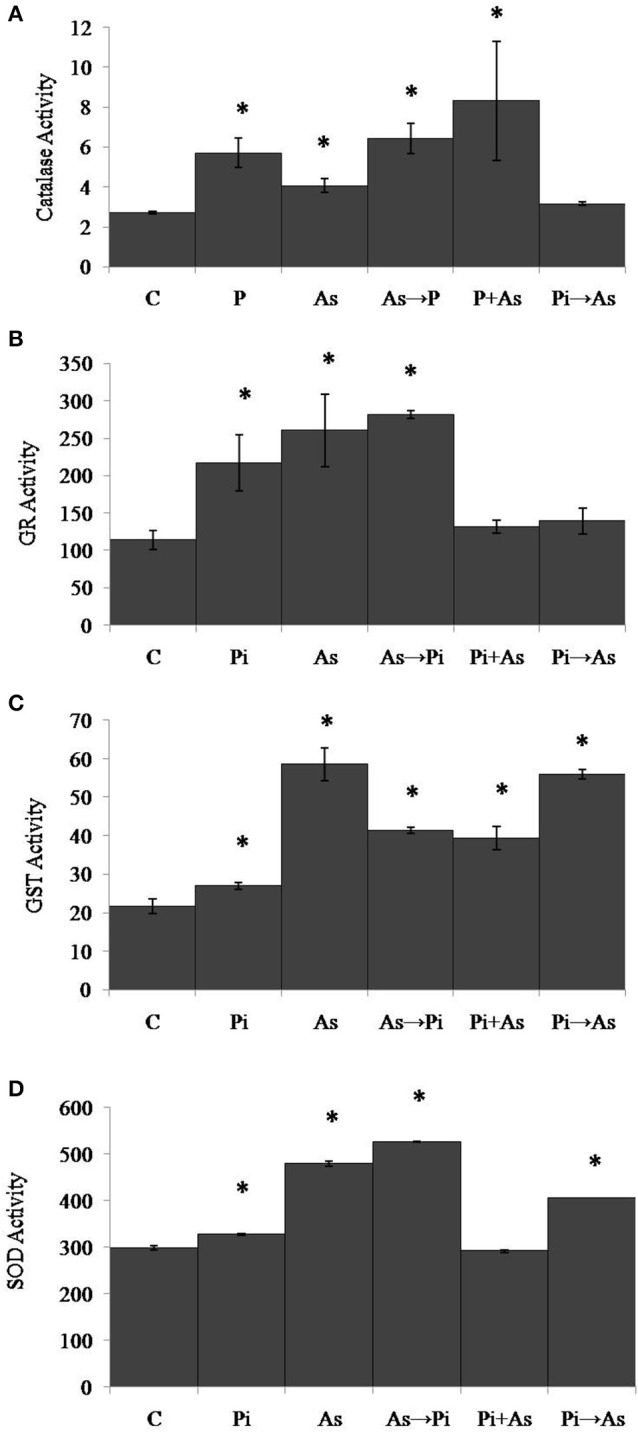
**Modulation of redox enzyme in rice shoot after treatment of ***P. indica*** and arsenic works as a defense against oxidative stress. (A)** CAT, **(B)** GR, **(C)** GST, and **(D)** SOD specific activities compared with those of control plants **(C)**; asterisks show values significantly different from those of the controls (*P* < 0.05). All experimental conditions were the same as described for Figure 1.

In the case of shoot, plants treated with arsenic show significant increase in CAT, GR, GST, and SOD activities and were found 1.5, 2.3, 2.5, and 1.6 folds respectively as compared to control plants (Figures 5A–D). However, GR activity was reduced up to 40% in the case of arsenic-treated plant shoot (Figure 5B). An increased CAT activity (2 fold) was observed in plants colonized with *P. indica* as compared to non-colonized plants (Figure 5A). Similarly, GR, GST, and SOD activities were found 1.9, 1.2, and 1.1 folds increased respectively (Figures 5B–D). In all three conditions of alternate and simultaneous treatment of arsenic and fungus, it was observed that CAT, GR, GST, and SOD were increased significantly as compared to control plants (*P* < 0.05) except SOD activity in case of simultaneous treatment of arsenic and endophyte (Figures 5A–D).

### *P. indica* reduces the arsenic load in rice plant

We compare the absorption of arsenic through the plant root in colonized and non-colonized state of the hydroponic culture. It was observed that pre-colonized plant root accumulated arsenic up to 26.22 ± 0.81 mg/g dry weight (40 fold increase) while non-colonized arsenic-treated plant root accumulates arsenic up to 0.65 ± 0.01 mg/g dry weight. The accumulation of arsenic in the shoot of pre-colonized plants was 0.039 ± 0.003 mg/g dry weight (55 fold decrease) and arsenic content in the shoot of non-colonized arsenic treated plants was 2.16 ± 0.44 mg/g dry weight. Interestingly, colonization alters the distribution of arsenic in the plant system and was mainly restricted to the root system of colonized plants (26.22 ± 0.81 mg/g dry weight) and a fraction of it translocated to shoot (0.039 ± 0.003 mg/g dry weight, Table 2). Results suggest a significant reduction in arsenic translocation from root to shoot in case of pre-colonized plant. The translocation factor is decreased to 1.48 × 10^−3^ in this treatment as compared to arsenic treated non-colonized plant where it was 3.32. A similar pattern was also observed in the case of simultaneous and alternate treatment of arsenic and endophyte in long-term experimental up to 25 dai (Table 2). Dithiozone assay was also done to visualize arsenic accumulation in the shoot. We observed the endophyte colonization restrict the arsenic distribution into the root, and shoot receives comparatively less arsenic than arsenic-treated plant and therefore shoot stained lighter (Supplementary Figure 2).

**Table 2:** **Fungal colonization and arsenic translocation**.

**S.No**.	**Sample**	**Arsenic content in root (mg/gm root biomass)**	**Arsenic content in shoot (mg/gm shoot biomass)**
1	Control	0.13 ± 0.055	0.03 ± 0.004
2	Pi	0.02 ± 0.003	0.08 ± 0.044
3	As	0.65 ± 0.011	2.16 ± 0.44
4	As → Pi	6.97 ± 1.246	0.21 ± 0.006
5	As+Pi	0.49 ± 0.009	1.34 ± 0.062
6	Pi → As	26.22 ± 0.81	0.039 ± 0.003

*Treated and control plants were analyzed for arsenic uptake and effect of fungal colonization on arsenic translocation to shoot. The amount of arsenic present in the plant root and shoot is expressed in mg per gm biomass*.

### *P. indica* can tolerate arsenic axenically

**Figure 6:**
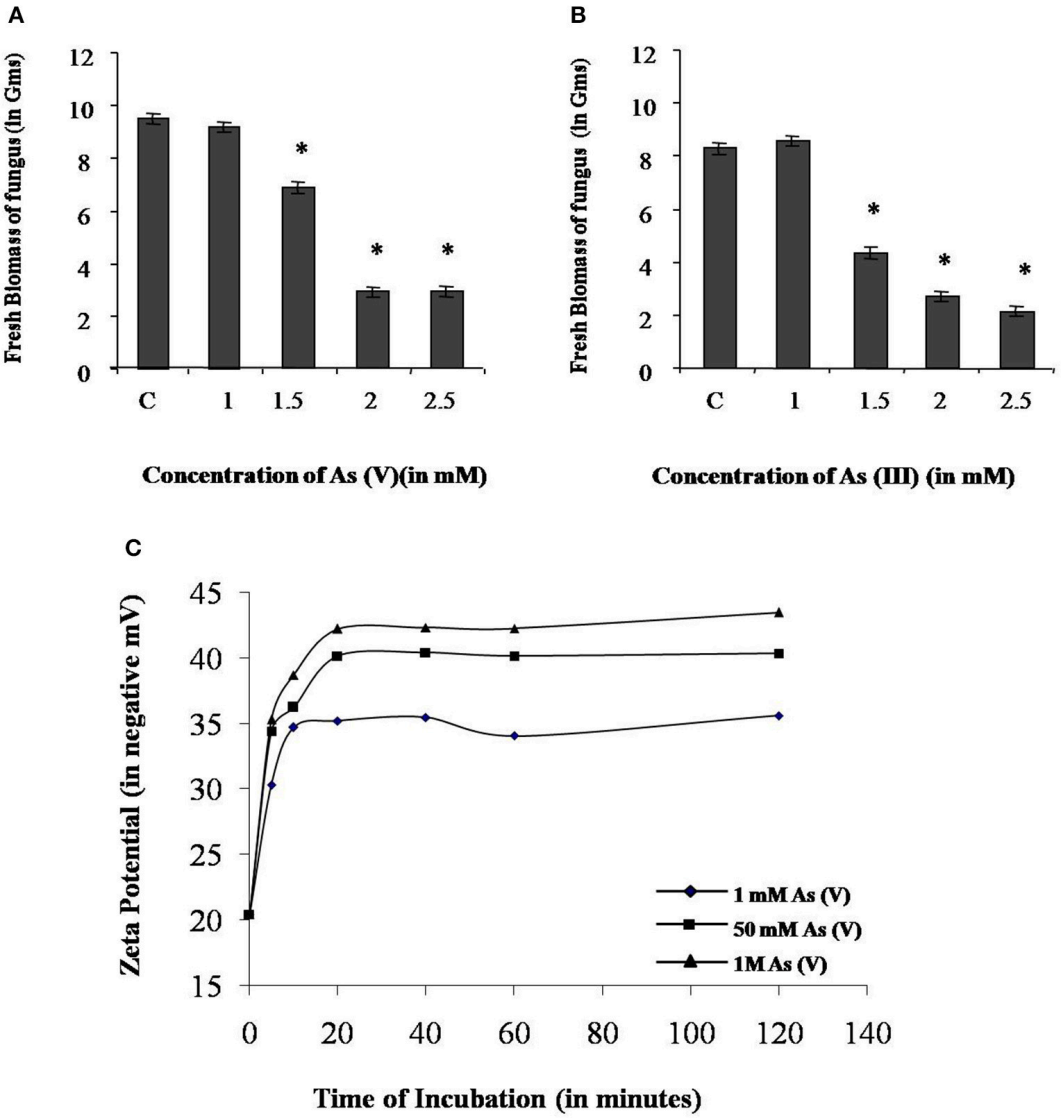
**Characterization of arsenic resistance by fungus ***P. indica***. (A)** Growth analysis of fungus at different concentration of sodium arsenate (As V). **(B)** Growth analysis of fungus at different concentration of sodium arsenite (As III). **(C)** Analysis of change in zeta potential on the cell wall due to adsorption of arsenic at 1 mM, 50 mM, and 1 M sodium arsenate (As V). Asterisks show values significantly different from those of the controls (*P* < 0.05).

In the axenic culture, *P. indica* is able to tolerate both sodium arsenate (As V) and sodium arsenite (As III) up to 1 mM. However, the growth of fungus was reduced 30 and 50% in presence of 1.5 mM As V and As III respectively. At 2 and 2.5 mM concentration of arsenic the growth of fungus was reduced up to 70% (Figures 6A,B).

### *P. indica* adsorbs arsenic on cell wall and accumulates in vacuoles

**Figure 7:**
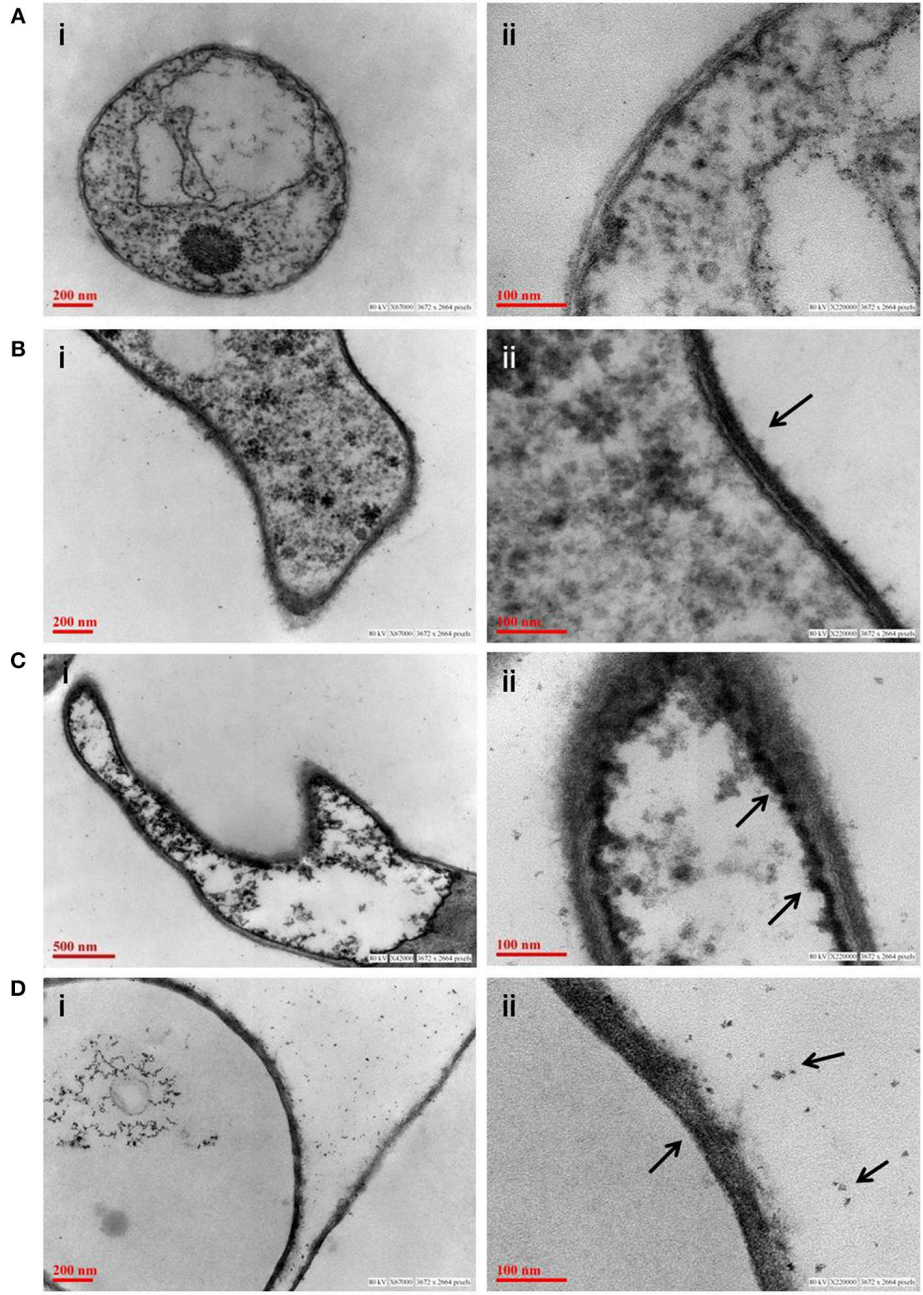
**Transmission electron micrograph of fungus ***P. indica*** showing arsenic adsorption, accumulation and precipitation. (A)** Untreated fungus; **(B)** accumulation of arsenic on cell wall of fungus (marked with arrows) treated with sodium arsenate (As V); **(C)** accumulation of arsenic on in vacuole of fungus (marked with arrows) treated with sodium arsenate (As V); **(D)** synthesis of insoluble precipitates of arsenic (marked with arrows) on cell wall of fungus treated with sodium arsenite (As III).

**Figure 8:**
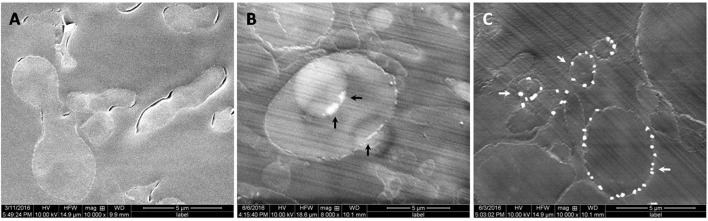
**Scanning electron micrograph of fungus ***P. indica*** showing arsenic adsorption, accumulation, and precipitation. (A)** Untreated fungus; **(B)** accumulation of arsenic in vacuoles and on cell wall of fungus (marked with black arrows) treated with arsenic (100 μM As); **(C)** Synthesis of insoluble precipitates of arsenic (marked with white arrows) on cell wall of fungus treated with arsenic (500 μM As).

Adsorption study was done to show the ability of the cell wall to capture dissolved toxic arsenic from the media. It was observed that cell wall was able to adsorb arsenic in a non-linear fashion and suggesting increasing the concentration satiate the As adsorption (Figure 6C). Adsorption of arsenic was 28.8 and 20% at 0.1 and 2 mM arsenic respectively. Arsenic adsorption saturated at 4 mg per gram cell wall (Table 3). To confirm this TEM analysis of the fungal cell was done to localize arsenic in the cell. We noticed an electron dense layer on the cell wall and vacuoles in cells treated with arsenic when treated with 100 μM arsenic salts (Figures 7B,C). Further, we also detected insoluble precipitates of arsenic in both TEM and SEM analysis (Figure 8). EDAX analysis also reveals the vacuolar content and precipitation of arsenic around cell wall, contains up to 0.53 and 3% arsenic respectively (Supplementary Figures 3, 4 and Supplementary Tables 1, 2). Some marked modification also observed in the arsenic-treated cell such as vacuolization, cell expansion, and changes in cell wall (Figures 7, 8).

**Table 3:** **Adsorption studies of Arsenic on the cell wall**.

**S.No**.	**Sample Treatment (for 60 min.)**	**Amount of Arsenic adsorbed (per gram fresh weight of cell wall)**
1	0.1 mM As (V)	0.216 ± 0.06 mg
2	1 mM As (V)	1.694 ± 0.43 mg
3	2 mM As (V)	2.913 ± 0.23 mg
4	50 mM As (V)	3.873 ± 0.16 mg
5	1 M As (V)	4.221 ± 0.13 mg

*One gram of cell wall was incubated in 100 ml of arsenic solution for 60 min. and cell wall material was washed with 1X PBS three times. Adsorption analysis was done in triplicates independently*.

## Discussion

In the natural habitat, almost all terrestrial plants make an association with arbuscular mycorrhizal fungi (AMF) except a limited number of plant (such as members of Amaranthaceae, Chenopodiaceae, Cyperaceae, Juncaceae, Proteaceae or with lupines, and Brassicaceae), resulting in a range of beneficial consequences to their hosts. AMF improves nutritional status, impart resistance to soil borne pathogens and tolerance to salt, drought, and heavy metals (Kumar et al., 2009, 2011; Orlowska et al., 2012; Jogawat et al., 2013; Spagnoletti and Lavado, 2015). Previous works have demonstrated the benefits of P. *indica* over AMF and its application (Varma et al., 1999; Kumar et al., 2009; Yadav et al., 2010; Jogawat et al., 2013). In the present study, we have established the role of *P*. *indica* in the bioprotection against arsenic toxicity and restricted accumulation of arsenic in host plant. In this study, we found that treatment of arsenic to plant reduces overall biomass, root, and shoot growth in rice and hampers the overall plant growth. *P. indica* colonization in plants helps in recovery from the hampered growth arising due to arsenic toxicity. Growth recovery and increase in biomass indicating the detoxification capacity of the *P. indica*. We have shown that the recovery in plant growth from arsenic stress in presence of *P. indica* is due to reduced arsenic translocation to shoot, immobilization of arsenic into root, storage of arsenic in fungal cells, and reprogramming the host cell redox status.

Our result showed that arsenic does not affect the colonization in the rice root initially but induces hyper-colonization in the arsenic-treated plant root without hampering physical traits (Figure 1 and Table 1). Colonization of *P. indica* in rice plants significantly lowers the susceptibility to arsenic. *P. indica* improved plant growth, biomass, and root integrity even in presence of toxic dosage of arsenic. *P. indica* induces abiotic-tolerance and arsenic resistance in hosts (Figures 1, 2). It was observed that the arsenic treatment reduces the chlorophyll b only but no change was observed for chlorophyll a and carotenoids. Endophyte colonization helps the plants in recovery from arsenic-induced chlorosis by enhancing the chlorophyll b contents in the arsenic-treated plants (Figure 3A). However, no improvement in anthocyanin content was observed under the influence of fungal colonization (Supplementary Figure S5). Further, we observed a significant higher proline content in the colonized plants (both treated and untreated) than the arsenic-treated and control plants (Figure 3B). It has been observed that endophyte induces proline accumulation in the plant (Jogawat et al., 2013) which help to overcome the adverse effects of ROS by pro-pro cycle and reduces the damage to plant (Liang et al., 2013; Signorelli et al., 2014). We observed 1.9 times higher accumulation of hydrogen peroxide (H_2_O_2_) in arsenic-treated rice plant than control (Figure 3C) as shown previously (Nath et al., 2014). At the same time arsenic treatment to rice plant significantly induces CAT (Figures 4A, 5A) and GR (Figures 4B, 5B) activity but a reduction in GST (Figure 4C) and SOD (Figure 4D) activity was observed, which may impair the antioxidative system of the plant and results in the accumulation of H_2_O_2_. *P. indica* colonization significantly reduces H_2_O_2_ content in arsenic treated plants but was still higher than the control plants. However, untreated colonized plants do not show any significant accumulation of H_2_O_2_. It is evident that an established mutualism between plant and fungus *P. indica* suppresses the accumulation of H_2_O_2_ in colonized plant (Schäfer et al., 2007) and this may be a possible explanation of reduction of H_2_O_2_ content in treated plants colonized by fungus. *P. indica* strongly induces CAT, GR, and restore the SOD activities in treated colonized plant which help plant to reduce reactive oxygen species and minimize damage caused by arsenic-mediated oxidative stress. It is suggesting that *P. indica* induces a systemic redox reprogramming leads to induction of resistance in plant and helping plant to recover its biomass and normal proliferated roots.

**Figure 9:**
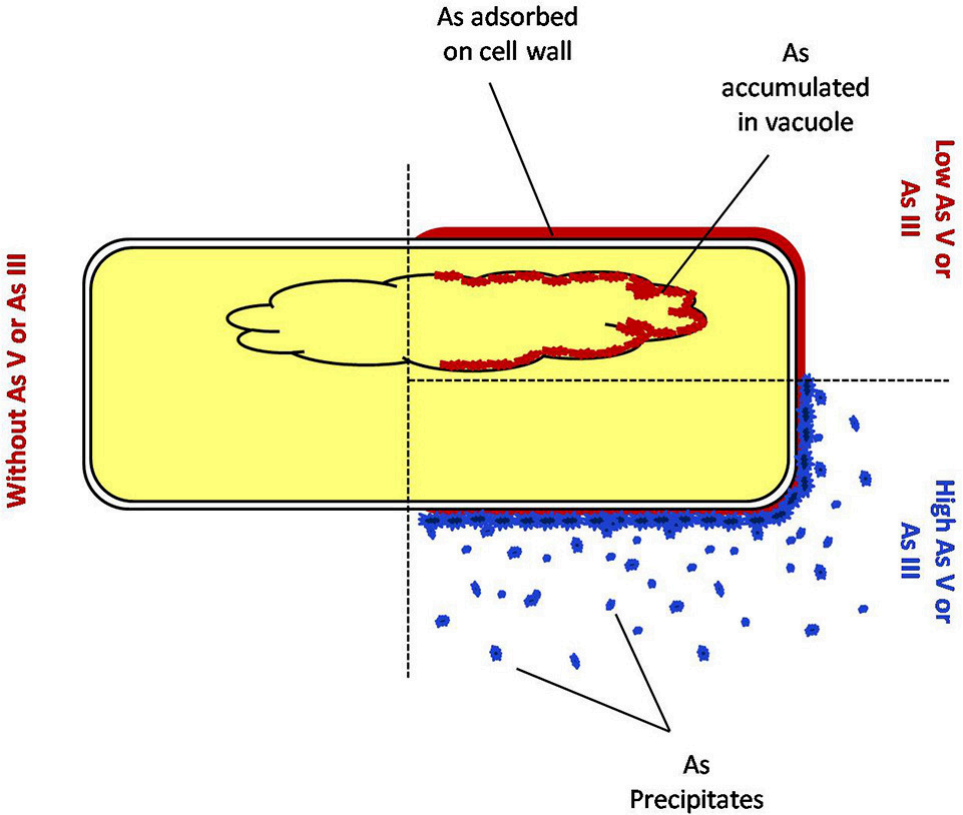
**Mechanism of detoxification of arsenic by fungus endophytic fungus ***P. indica*****.

Interestingly, this was observed that a lower amount of arsenic was accumulated in shoot in all the cases of *P. indica* colonized treated plants as compared to arsenic-treated plants (Table 2 and Supplementary Figure 1) and colonization of endophyte in the root reduces the translocation factor drastically in the plant (Marchiol et al., 2004). These results suggest that reduction in arsenic accumulation in shoot is either due to *P. indica* mediated induction of immobilization of arsenic in plant root or *P. indica* itself function as an arsenic-screen and restricting arsenic to be translocated to the shoot. We performed assays and observed that there is no inhibitory effect on fungal growth of arsenic at a concentration of 1 mM which revealed that endophyte has tolerance to arsenic toxicity (Figures 6A,B). Further, we observed that fungal cell wall has a great capacity to adsorb arsenic on the cell wall (Figures 6C, 7) and which is up to 4 mg per gram fresh weight of cell wall (Table 3). This result suggesting that the induced arsenic-tolerance imparted by the fungus is due to selective clearance of arsenic by the extra-radical hyphae of fungus from the media and this is why root receives a large amount of arsenic while a small fraction of it can mobilize to shoot. In colonized state, extra-radical hyphae may apply alternate strategy to remove a large amount of arsenic which is not possible by adsorption only, as it requires a large amount of fungal cell to adsorb arsenic. This hypothesis was supported by one of our observation in which arsenic exposure induces a vacuolization (formation of multiple or larger vacuoles) in *P. indica* and these vacuoles store a significant amount of arsenic (Figures 7C, 8). Therefore, it is suggested that the process of compartmentalization of arsenic may be a unique mechanism which may also play role in detoxification of the arsenic during colonization and that has to be studied. In a previous study a metal tolerant fungus, *Exophiala pisciphila*, (H93) was shown to accumulated over 5% Cadmium (Cd) of its dry weight intracellularly (Zhang et al., 2008) and enhances colonized maize plant's tolerance to Lead, Zinc, and Cd (Li et al., 2011). Similarly, our study has also revealed that the endophyte imparts tolerance to arsenic using not only vacuolar entrapment and cell wall adsorption but it also precipitates arsenic out side of the fungal cell (Figure 8). We observed arsenic precipitation around cell wall containing arsenic up to 3% and this may also play important role in the immobilization of arsenic. The process of immobilization of arsenic into the root is mainly due to adsorption, compartmentalization into the vacuole and transforming arsenic into precipitates (Figure 9). These fungal mediated processes reduce the amount of soluble arsenic around colonized root and therefore minimize the arsenic transportation and accumulation in the shoot. The bioprotective and arsenic screening nature of *P. indica* can be utilized in the agricultural field contaminated with arsenic.

The fungal mediated tolerance against arsenic in plant is led by decrease in free or available arsenic in the surroundings with an additive effect on the anti-oxidative metabolite and anti-oxidative enzyme system of the plant. The plants possess a potent cellular environment to deal with arsenic mediated toxicity such as reactive oxygen species (ROS) in which both the anti-oxidative metabolites and enzyme systems play a crucial role. However, the susceptible varieties of plant were unable to induce both of these systems (Rai et al., 2011; Shankar et al., 2016). It was interesting to note that in the present study, both parameters enhanced under arsenic stress in the colonized plants (sensitive variety; IR 64) which does not negatively affect biomass yield of rice. Further, the capacity of immobilization of arsenic by *P. indica* reduces the arsenic load in rice shoot makes crop safer to consume. Exploitation of axenically culturable endophytic fungus *P. indica* may not only add value to modern crop-growing strategies in arsenic contaminated area but may also serve as a model system to study molecular traits of metal resistance in plant-microbe interaction and bioremediation.

## Author contributions

The work presented here was carried out in collaboration between all authors. Fungal colonization analysis, Antioxidant enzyme activities in rice plants and Estimation of Chlorophyll and Proline estimation were done by SM and MK. Dithizone test and Estimation of H_2_O_2_ production was done by JShukla and SM. Arsenic adsorption analysis was done by MK. Transmission Electron Microscope, Scanning Electron Microscope and EDAX analysis were done by JShukla, ASK, MK, JShankar, NA, and PNS. Confocal imaging was done by SM and RN. Arsenic accumulation studies in plants were done by SM. Arsenic estimation in plants was done by KM and SKR. MK defined the research theme. MK coordinated the project and wrote the manuscript. All authors have contributed to, seen and approved the manuscript.

### Conflict of interest statement

The authors declare that the research was conducted in the absence of any commercial or financial relationships that could be construed as a potential conflict of interest.
