# Supplementary material for: Endophytic Fungi Piriformospora indica Mediated Protection of Host from Arsenic Toxicity
Source: Front Microbiol. 2017 May 10;8:754. doi: 10.3389/fmicb.2017.00754 (PMC5423915; doi:10.3389/fmicb.2017.00754)
Supplement: Supplementary file 1 [file DataSheet1.PDF]

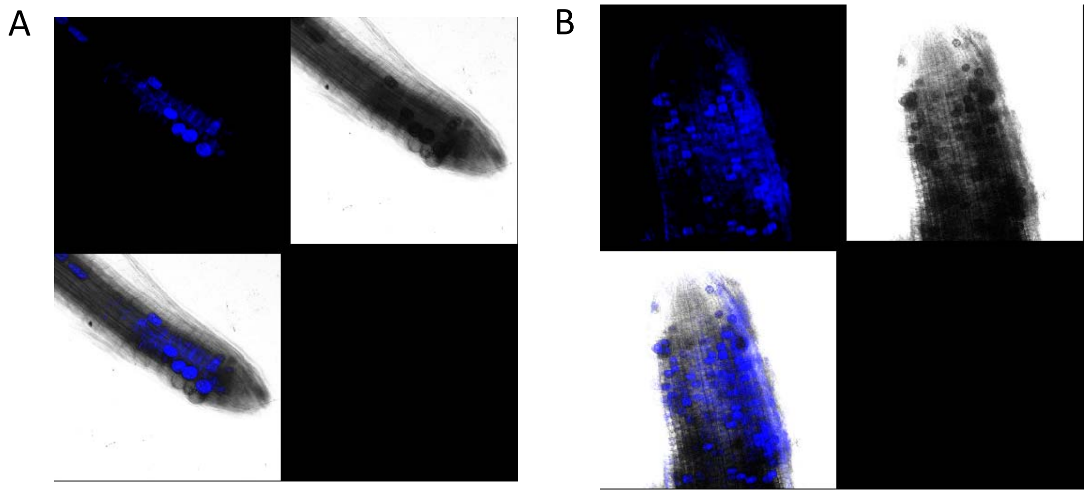

Supplementary Figure. S1. Confocal images of *P. indica* in un-treated root (A) and treated with arsenic (B). Calcofluor staining gives a blue colour to mycelium and spore of fungus. Figures showing a higher no. of spores present in the root treated with arsenic than un-treated root.

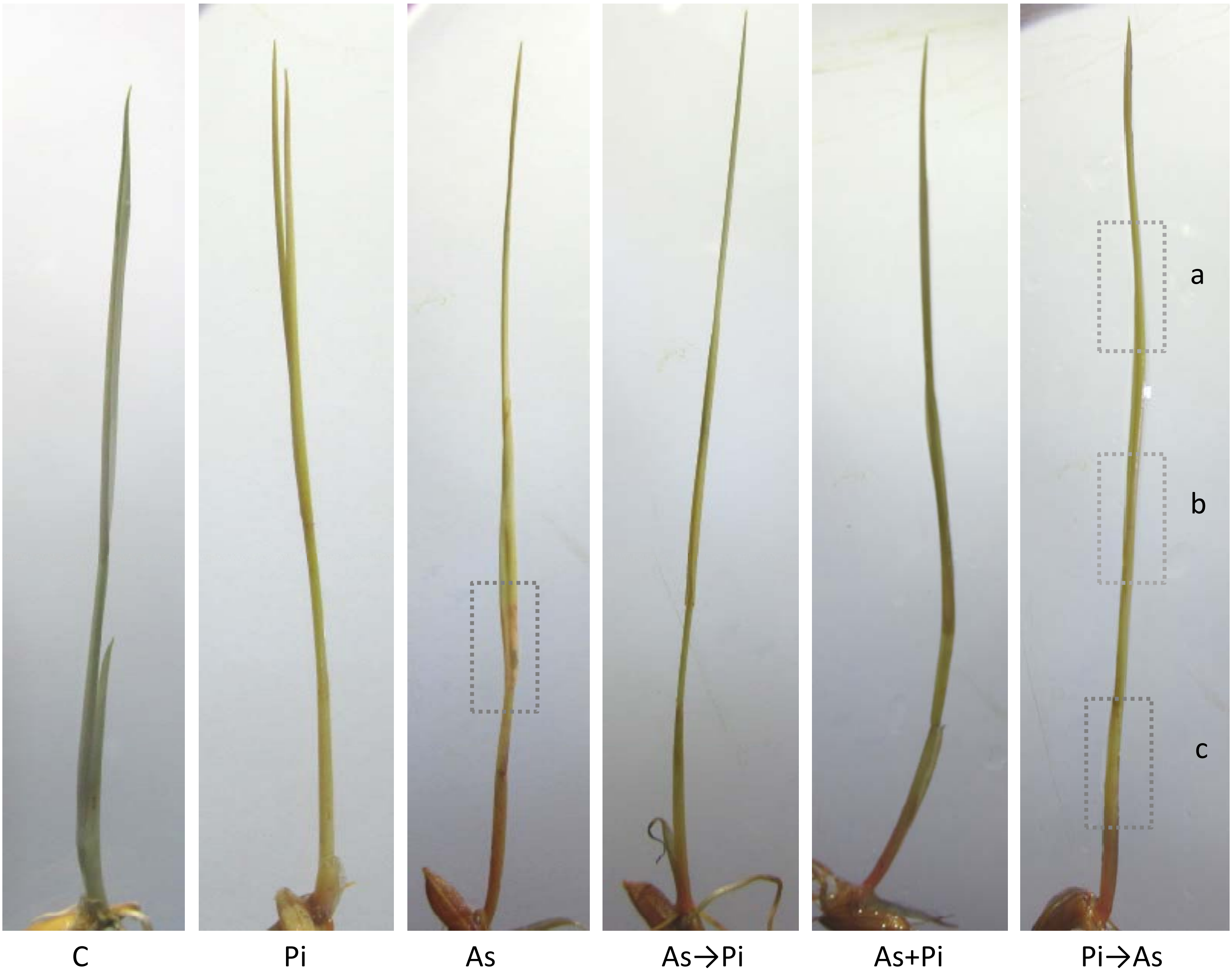

Supplementary Figure. S2. Localization of arsenic in rice plant. Dithizone assay was done to stain the deposition of arsenic in the plant shoot. Red brown patches shown by arrows in the arsenic treated plant shoot (As) is arsenic deposition in plant (in box view) while no such deposition were observed in (Pi→As) plant (in box view a, b and c). All experimental conditions were the same as described for Fig. 1.

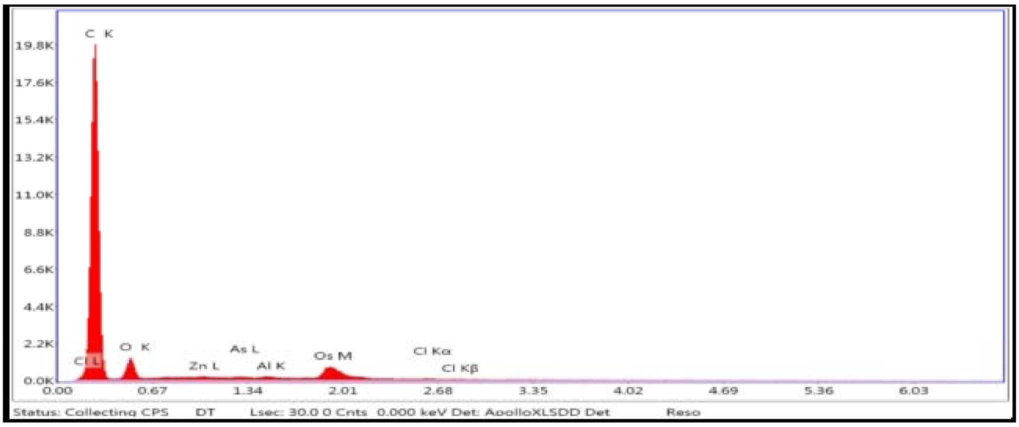

Element	Weight %	Atomic %	Net Int.	Net Int. Error
C K	83.1	90.91	4228.09	0
O K	10.27	8.44	288.58	0.01
ZnL	0.32	0.06	12.69	0.18
AsL	0.3	0.05	11.59	0.25
AlK	0.16	0.08	15.08	0.25
OsM	5.68	0.39	176.72	0.03
ClK	0.17	0.06	8.42	0.56

Supplementary Figure S3. EDAX analysis of vacuolar content of fungus *P. indica*

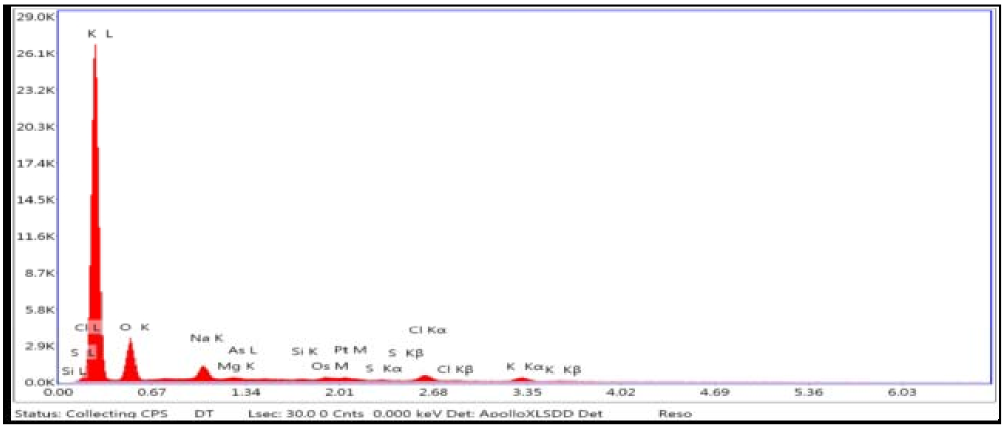

Element	Weight %	Atomic %	Net Int.	Net Int. Error
O K	45.69	66.92	779.57	0.01
NaK	14.75	15.04	326.11	0.02
MgK	1.4	1.35	40.01	0.09
AsL	3	0.94	35.38	0.1
SiK	1.39	1.16	40.3	0.1
OsM	6.62	0.82	68.34	0.07
PtM	6.77	0.81	61.24	0.07
S K	1.14	0.83	23.68	0.17
ClK	9.77	6.46	167.59	0.03
K K	9.47	5.67	113.14	0.04

Supplementary Figure S4. EDAX analysis of content of precipitate around cell wall of fungus *P. indica*

Composition of minimal Media:

SI Table 1. Composition of Minimal Media for fungus *P. indica*.		
S.No.	Constituents	Composition
1.	Glucose	10.0 gm/L
2.	Peptone	2.0 gm/L
3.	Yeast extract	1.0 gm/L
4.	$NaNO_3$	6.0 gm/L
5.	KCl	0.52 gm/L
6.	$MgSO_4$. $7H_2O$	0.52 gm/L
7.	KH_2PO_4	13.6 mg/L*
8.	$ZnSO_4$. $7H_2O$	22.0 mg/L
9.	H_3BO_3	11.0 mg/L
10.	$MnCl_2$. $4H_2O$	5.0 mg/L
11.	$FeSO_4$. $7H_2O$	5.0 mg/L
12.	$CoCl_2$. $6H_2O$	1.6 mg/L
13.	$CuSO_4$. $5H_2O$	1.6 mg/L
14.	$(NH_4)_6$ Mo_7O_{27}. $4H_2O$	1.1 mg/L
15.	Na_2EDTA	60.0 mg/L
16.	Biotin	0.5 mg/L
17.	Nicotinamide	5.0 mg/L
18.	Pyridoxal phosphate	1.0 mg/L
19.	Amino benzoic acid	1.0 mg/L
20.	Riboflavin	2.5 mg/L
21.	pH	5.8

Composition of Hydroponics Solution:

SI Table 2. Composition of Hydroponic solution for rice with some modification*(Kamachi et al. 1991).		
S. No.	Chemical	Final Conc.
1.	NH4NO3	1 mM
2.	NaH2PO4.2H2O	0.1 mM*
3.	K2SO4	0.3 mM
4.	CaCl2.2H2O	0.2 mM
5.	MgCl2 .6H2O	0.4 mM
6.	Fe-EDTA	45 mM
7.	H3BO3	50 mM
8.	MnSO4.5H2O	9 mM
9.	CuSO4.5H2O	0.3 mM
10.	ZnSO4.7H2O	0.7 mM
11.	Na2MoO4.2H2O	0.1 mM
12.	pH	5.7
