# Supplementary material for: Endophytic Fungi Piriformospora indica Mediated Protection of Host from Arsenic Toxicity
Source: Front Microbiol. 2017 May 10;8:754. doi: 10.3389/fmicb.2017.00754 (PMC5423915; doi:10.3389/fmicb.2017.00754)
Supplement: Supplementary file 2 [file Image1.pdf]

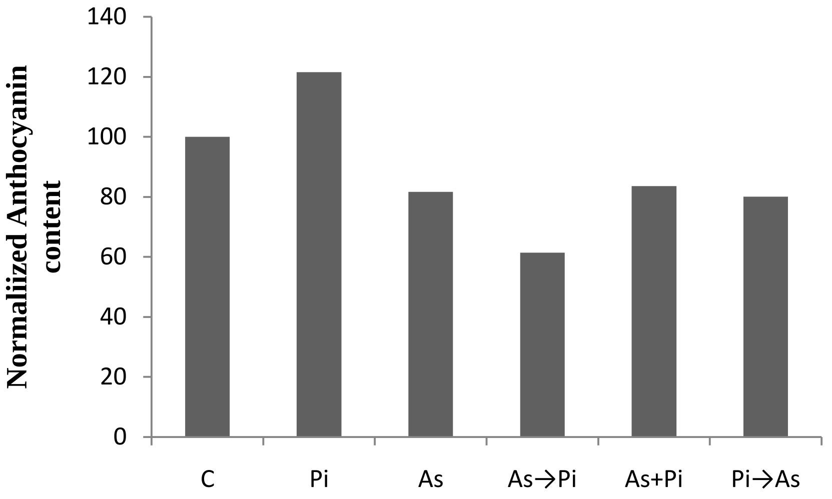

Figure S5. Impact of arsenic on Anthocynin content. Anthocynin content in the plants alternate and simultaneously treated with P. indica and arsenic. All experimental conditions were the same as described for Figure 1.
